# Crystal structures of VIM‐1 complexes explain active site heterogeneity in VIM‐class metallo‐β‐lactamases

**DOI:** 10.1111/febs.14695

**Published:** 2018-11-23

**Authors:** Ramya Salimraj, Philip Hinchliffe, Magda Kosmopoulou, Jonathan M. Tyrrell, Jürgen Brem, Sander S. van Berkel, Anil Verma, Raymond J. Owens, Michael A. McDonough, Timothy R. Walsh, Christopher J. Schofield, James Spencer

**Affiliations:** ^1^ School of Cellular and Molecular Medicine University of Bristol UK; ^2^ Institute of Infection and Immunity Cardiff University UK; ^3^ Department of Chemistry University of Oxford UK; ^4^ Oxford Protein Production Facility UK Rutherford Appleton Laboratory Oxfordshire UK

**Keywords:** antibiotic resistance, carbapenem, metallo‐β‐lactamase, VIM, X‐ray crystallography

## Abstract

Metallo‐β‐Lactamases (MBLs) protect bacteria from almost all β‐lactam antibiotics. Verona integron‐encoded MBL (VIM) enzymes are among the most clinically important MBLs, with VIM‐1 increasing in carbapenem‐resistant Enterobacteriaceae (*Escherichia coli*,* Klebsiella pneumoniae*) that are among the hardest bacterial pathogens to treat. VIM enzymes display sequence variation at residues (224 and 228) that in related MBLs are conserved and participate in substrate binding. How they accommodate this variability, while retaining catalytic efficiency against a broad substrate range, has remained unclear. Here, we present crystal structures of VIM‐1 and its complexes with a substrate‐mimicking thioenolate inhibitor, ML302F, that restores meropenem activity against a range of VIM‐1 producing clinical strains, and the hydrolysed product of the carbapenem meropenem. Comparison of these two structures identifies a water‐mediated hydrogen bond, between the carboxylate group of substrate/inhibitor and the backbone carbonyl of the active site zinc ligand Cys221, that is common to both complexes. Structural comparisons show that the responsible Cys221‐bound water is observed in all known VIM structures, participates in carboxylate binding with other inhibitor classes, and thus effectively replicates the role of the conserved Lys224 in analogous complexes with other MBLs. These results provide a mechanism for substrate binding that permits the variation at positions 224 and 228 that is a hallmark of VIM MBLs.

**Enzymes:**

EC 3.5.2.6

**Databases:**

Co‐ordinates and structure factors for protein structures described in this manuscript have been deposited in the Protein Data Bank (www.rcsb.org/pdb) with accession codes 5N5G (VIM‐1), 5N5H (VIM‐1:ML302F complex) and 5N5I (VIM‐1‐hydrolysed meropenem complex).

**Abbreviations**
MBL: metallo‐β‐lactamase
VIM: Verona Integron‐encoded MBL

## Introduction

Antibiotic resistance is of immediate and growing concern to global public health 1. Resistance in opportunistic Gram‐negative bacterial pathogens is of pressing importance as these organisms cause an increasing frequency of healthcare‐associated infections in immunocompromised individuals and treatment options are limited by both intrinsic and acquired resistance mechanisms 2. β‐Lactams, which continue to form over half of the global antibacterial market 3, remain key agents for treatment of such infections, with the carbapenems in particular rapidly supplanting third‐generation cephalosporins as first‐choice drugs. In pathogens such as the Enterobacteriaceae (e.g. *Escherichia coli*;* Klebsiella pneumoniae*) or nonfermenting organisms (e.g. *Pseudomonas aeruginosa*;* Acinetobacter baumannii*) production of β‐lactamases that inactivate β‐lactams via hydrolysis of the β‐lactam ring 4, is the major form of β‐lactam resistance 5. Over 1300 such enzymes have now been identified in isolates of diverse clinical origin 6. While the spectrum of activity against different β‐lactam classes varies between enzymes, carbapenem‐hydrolysing β‐lactamases are attracting increasing attention due to the efficiency with which they hydrolyse most β‐lactam classes and the growing frequency with which they are isolated from patients 7.

β‐Lactamases comprise two distinct groups – serine (SBL) and metallo‐ (MBL) β‐lactamases – which differ in their structure and catalytic mechanisms 8. Clinically available inhibitors (e.g. clavulanic acid, avibactam) are active against many, though not all, SBLs but all are ineffective against the MBLs 9. In addition, while carbapenems effectively inhibit most SBLs 10, all known MBLs effectively hydrolyse these antibiotics 11. In consequence, MBL dissemination poses a particular challenge to the continued effectiveness of β‐lactams, especially carbapenems, against Gram‐negative bacteria.

Metallo‐β‐lactamases possess a conserved protein fold that forms an αββα sandwich with the active site zinc centre found in a central groove on one edge of the two β‐sheets 12. Based upon differences in sequence and active site structures, the MBLs are divided into three subgroups (B1, B2 and B3 13) with the most clinically relevant IMP, VIM and NDM enzymes 14 contained within the subclass B1. The B1 active site binds two zinc ions, located respectively in the (usually tetrahedral) Zn1 site formed by the conserved histidines 116, 118 and 196; and the Zn2 site comprising Asp120, Cys221 and His263 15. (The standard MBL numbering scheme 16 is used throughout.) It is generally accepted that B1 enzymes require both zinc sites for maximal catalytic efficiency 17, 18.

The VIM (Verona Integron‐encoded MBL) enzymes are one of the most widely distributed MBL families and are a leading cause of carbapenem failure, particularly in *P. aeruginosa* 19. *bla*_VIM‐1_ was identified in 1999 as a chromosomally encoded gene on a cassette within a class I integron in a *P. aeuruginosa* clinical isolate from Italy 20. Subsequently, VIM enzymes have been identified on ‘multiresistance’ plasmids from multiple Gram‐negative species; over 50 different VIM variants have now been identified 21. Of these, VIM‐1 is noteworthy as it is most frequently encountered in the Enterobacteriaceae (*K. pneumoniae* and *E. coli* 22); most other VIM types are primarily associated with the less common nonfermenting pathogens, in particular *P. aeruginosa*. VIM (and other B1) MBLs have a broad spectrum of activity encompassing penicillins, cephalosporins and carbapenems (23 and references therein).

Sequence identity between VIM variants ranges from ~ 75% to > 99%, with VIM‐1 and VIM‐2 progenitors of two dominant phylogenetic clusters and VIM‐7 as a uniquely divergent variant 23. Sequence substitutions at residues 224 and 228 are a hallmark of VIM variants and of particular interest as these positions interact with β‐lactam substrates in other MBLs 24, 25. Although several crystal structures of VIM enzymes are now available 24, 26, 27, 28, including of the VIM‐1 point variants VIM‐4 29, and VIM‐26 25, structures of the progenitor enzyme VIM‐1 have not previously been reported. Notably, from both inhibitor design and mechanistic perspectives, there is as yet no structural information describing how any VIM enzyme binds β‐lactams. Here, we focus on structural investigations of VIM‐1 and its interactions with a non‐β‐lactam inhibitor that mimics β‐lactam binding, ML302F 30, as well as a clinically relevant carbapenem substrate, meropenem. Our results reveal how substrate/inhibitor binding by VIM enzymes is tolerant of substitutions at sequence positions that in other MBLs make interactions, notably those involving the β‐lactam carboxylate, that are crucial for activity.

## Results

### Crystal structure of VIM‐1

**Figure 1:**
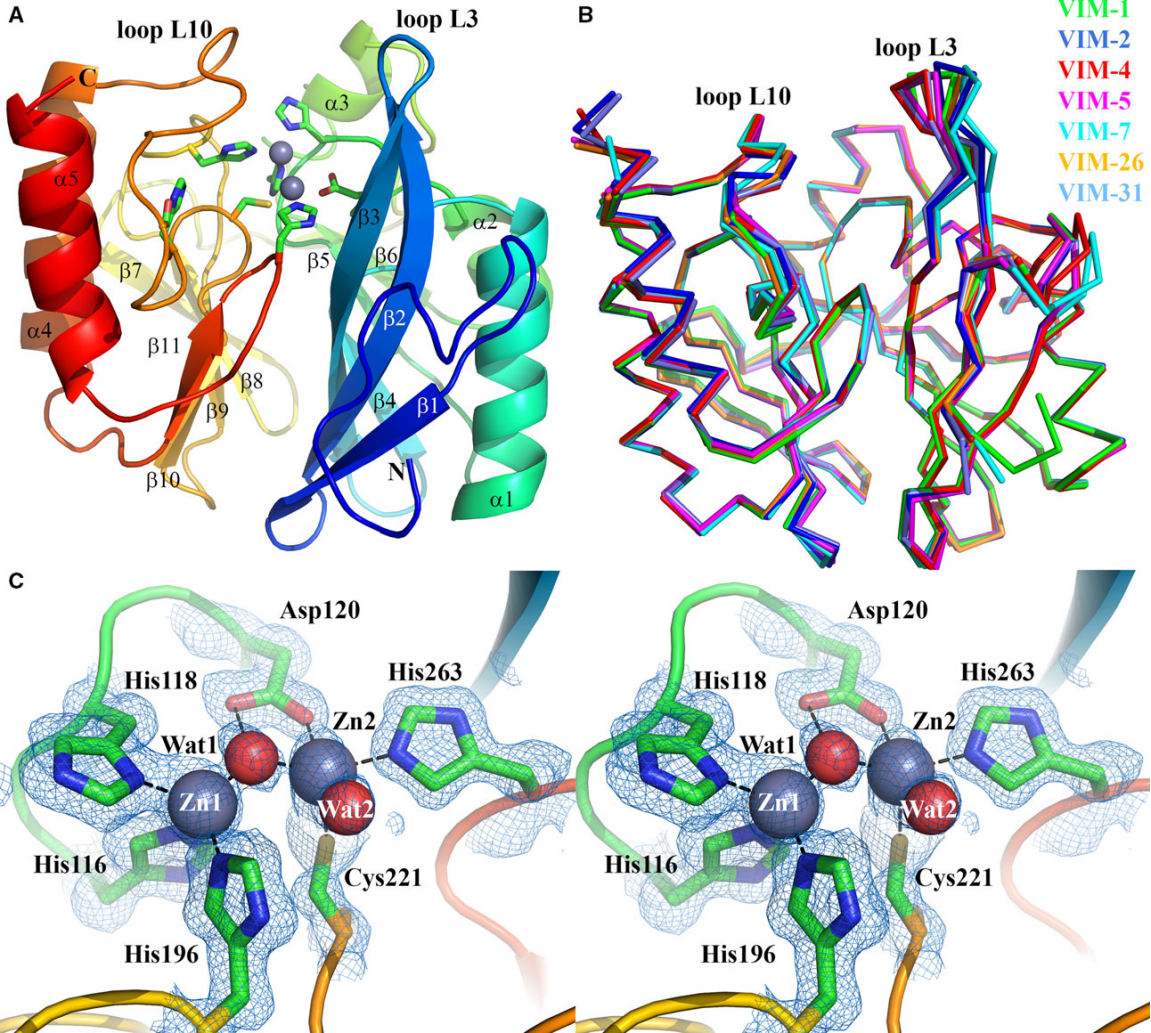
Crystal structure of VIM‐1. (A) View of the crystal structure of di‐zinc VIM‐1 showing the overall fold and location of active site. Protein main chain is colour‐ramped from blue (N terminus) to red (C terminus). Active site residues are rendered as sticks; zinc ions as grey spheres. Secondary structure elements are labelled. (B) Superposition of di‐zinc VIM‐1 with structures of other VIM enzymes (VIM‐2, pdb 4BZ3; VIM‐4, pdb 2WHG; VIM‐5, pdb 5A87; VIM‐7, pdb 4D1T; VIM‐26, pdb 4UWO; VIM‐31, pdb 4FR7; coloured as shown). (C) Stereoview of di‐zinc VIM‐1 active site. Electron density shown is 2|*F*_o_| − |*F*_c_| Φ_calc_ contoured at 1.5 σ. This Figure was created using pymol (www.pymol.org).

Crystals of VIM‐1 formed after several days’ incubation at 20 °C and diffracted to near atomic resolution (1.29 Å; Table 1). Initial analysis showed these to be of space group P 2_1_ with a solvent content of 41.4%, indicating one molecule in the asymmetric unit. Structure solution by molecular replacement, and subsequent model building, were straightforward, with the complete chain traced without interruption between residues Gly25 and His293 giving a final model containing 233 residues. The structure displays the overall αβ/βα fold of the MBL superfamily, with the binuclear zinc centre that forms the active site situated in a shallow groove formed by the interface of the two αβ domains (Fig. 1A). Two extended loops, termed L3 (residues 60–66) and L10 (residues 221–241) border the active site. Superposition of the VIM‐1 structure with those of other VIM family members using pdbefold 31 yields RMSD values for Cα‐atoms of between 0.28 Å (VIM‐26, pdb 4UWP 25) and 0.83 Å (VIM‐31 (oxidized) pdb 4FSB 28) Å, demonstrating that only small differences are evident between the overall fold of VIM‐1 and other structurally characterized VIM variants (Fig. 1B). These differences are largely localized to the L3, and to a lesser extent the L10, loops.

**Table 1:** Data collection and refinement statistics

	Native VIM‐1	VIM‐1:ML302F	VIM‐1:Meropenem
Data collection			
X‐ray source	DLS (I04)	DLS (I02)	DLS (I24)
Wavelength (Å)	0.9795	0.9795	0.9686
Space group	P2_1_	P2_1_	P2_1_
Cell dimensions			
*a*,* b*,* c* (Å)	39.76, 67.94, 40.36	39.68, 67.65, 40.22	38.94, 68.08, 39.82
α, β, γ	90, 94.01, 90	90, 91.36, 90	90, 90, 90
Molecules/asymmetric unit	1	1	1
Resolution (Å)	40.26–1.29 (1.31–1.29)	28.58–1.30 (1.32–1.30)	27.84–2.20 (2.28–2.20)
No. of unique reflections	61 346	52 058	10 547
Redundancy	3.6 (3.4)	6.4 (6.1)	5.8 (5.7)
*R*_pim_	0.039 (0.326)	0.033 (0.201)	0.081 (0.240)
CC_1/2_	0.997 (0.763)	0.997 (0.881)	0.988 (0.870)
*I/σ(I)*	12.5 (2.9)	21.5 (7.9)	8.5 (4.2)
Completeness (%)	99.3 (98.7)	99.8 (99.4)	99.2 (98.6)
Refinement			
Resolution (Å)	34.25–1.29	28.58–1.30	27.84–2.20
No. of reflections	53 159	52 032	10 543
*R*_work_/*R*_free_	0.1445/0.1563	0.1545/0.1678	0.1526/0.2250
Atoms			
Protein	3432	3496	1755
Ligand	N/A	19	27
Zinc	3	2	3
Solvent	328	237	85
B‐factor (Å^2^)			
Protein	15.19	15.32	16.99
Ligand	N/A	15.96	39.13
Zinc	16.15	8.34	25.96
Solvent	29.62	28.42	22.35
RMSD			
Bonds (Å)	0.008	0.020	0.006
Angles (°)	1.262	1.672	1.076
PDB accession	5N5G	5N5H	5N5I

5% of reflections were set aside for R_free_ calculation. imosflm, xds, aimless and phaser mr were used for structure solution and phenix for refinement.

Metal ions observed in both metal sites were refined as zinc ions, with occupancies of 0.94 (Zn1) and 0.73 (Zn2), respectively. Although reduced occupancy was observed for the Zn2 site, the Cys221 ligand was refined as the fully reduced form. An additional zinc ion was present (occupancy 0.54) bound to a bicine buffer molecule at the protein surface. The two active site zinc ions are separated by a distance of 3.62 Å with Zn1 in a tetrahedral geometry and Zn2 showing octahedral coordination with one vacant position 32 (Fig. 1C). Two well‐defined water molecules are present in the active site: a bridging water/hydroxide (Wat1, B‐factor 14.65 Å^2^) positioned asymmetrically between the two zinc ions, lying closer (1.89 Å) to Zn1 than to Zn2 (2.18 Å); and a second water molecule (Wat2, B‐factor 21.67 Å^2^) apparently tightly (2.06 Å) bound to Zn2.

**Figure 2:**
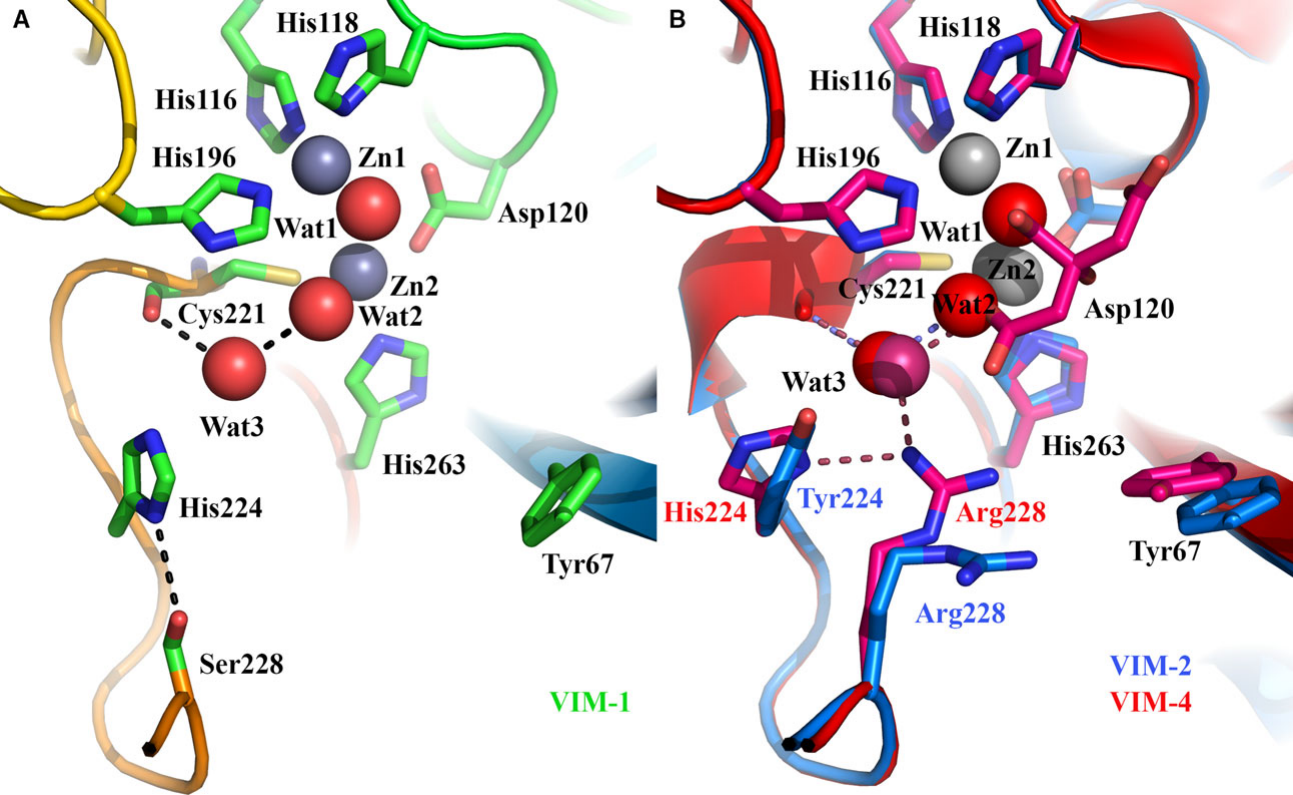
Comparison of VIM active sites. (A) Active site of VIM‐1, showing positions of His224 and Ser228 and location of Cys221‐bound water Wat3. Hydrogen bonding interactions are shown as dashed lines. (B) Active site superpositions of VIM‐2 (pdb 4BZ3, blue) and VIM‐4 (pdb 2WHG, red) showing variations at positions 224 and 228.

Functionally significant differences between VIM variants have been associated primarily with substitutions at positions 224 and 228 on loop L10 23, 26 where VIM‐1 possesses His and Ser residues, respectively (Fig. 2A). Notably, in our VIM‐1 structure His224 is oriented by a strong hydrogen bond (2.92 Å) between Oγ of Ser228 and Nδ1 of His224, such that the side chain imidazole ring forms one wall of the active site cleft (Fig. 2A). In contrast, in structures of enzymes such as VIM‐4, VIM‐7 and VIM‐31, His224 Nδ1 instead is H bonded to Nω1 of Arg228, apparently causing rotation of the His224 side chain to lie perpendicular to its position in VIM‐1 27, 28, 29. In VIM‐1, the orientation of the His224 side chain more closely resembles that of the phenolic ring of Tyr224 in VIM‐2 (Fig. 2B). Moreover, in the VIM‐2, ‐4, ‐7 and ‐31 structures, the side chain of Arg228 protrudes into the active site groove towards the side chain of Tyr67, which is located at the base of the L3 loop on the opposite side of the cleft. Thus, substitution of Arg228 for Ser in VIM‐1 (and in VIM‐26 25 where His224 is substituted by Leu) expands the active site cleft compared to other VIM enzymes of known structure.

### Inhibition of VIM‐1 by the thioenolate ML302F

Despite their sequence differences, and the growing clinical importance of VIM‐1, many studies of MBL inhibitors have used VIM‐2 as a representative VIM enzyme. Recently, we reported that the thioenolate hydrolysis product, ML302F, of the rhodanine ML302, is a potent (submicromolar) inhibitor of both VIM‐1 and VIM‐2 *in vitro* 30, and proposed that ML302F binding to MBLs mimics that of β‐lactam substrates/intermediates. Accordingly, we sought to extend these findings by studying the interactions of ML302F with VIM‐1 in producer bacteria and by X‐ray crystallography.

To investigate interactions of ML302F with VIM‐1 in bacteria, we determined minimal inhibitory concentrations (MICs) for the clinically important carbapenem antibiotic meropenem by broth microdilution for 27 VIM‐1 expressing *K. pneumoniae* and *E. coli* clinical strains in the presence and absence of 10 μg·mL^−1^ ML302F (Table 2). All 20 *K. pneumoniae* strains could be considered as resistant to meropenem according to either CLSI or EUCAST clinical breakpoints; however, only two of seven *E. coli* could be classed as resistant, with the rest showing meropenem susceptibility that, while substantially reduced compared to control, was still within the susceptible range. For 8 of 20 *K. pneumoniae* strains, the meropenem MIC in the presence of ML302F changes from resistant to susceptible according to EUCAST breakpoints, with a further six strains changing from resistant to intermediate susceptibility. Of the remaining six strains tested, five showed MIC reductions in one (twofold) to two (fourfold) dilutions, with one being unaffected. ML302F reduced the meropenem MICs of the two resistant *E. coli* strains to susceptible in one case and to intermediate in the other. In the five susceptible strains, reductions in at least two dilutions (fourfold or more) were observed in four cases, with one strain apparently unaffected. These results show that ML302F can potentiate the activity of meropenem against clinical isolates of VIM‐1 expressing Enterobacteriaceae, and thus that this compound can penetrate clinically relevant bacteria to act as an effective inhibitor of VIM‐1 in the bacterial host.

**Table 2:** Effect of ML302F on meropenem MICs for VIM‐1‐expressing enterobacteriaceae

Strain	Meropenem MIC (μg·mL^−1^)	Meropenem resistant/intermediate/susceptible (clinical strains; EUCAST^a^)	Meropenem MIC (μg·mL^−1^) + 10 μg·mL^−1^ ML302F	Meropenem resistant/intermediate/susceptible (clinical strains; EUCAST^a^)	Fold difference in MIC
*Escherichia coli* TOP1O (control)	≤ 0.25		≤ 0.25		0
*Klebsiella pneumonia*e NCTC 5055 (control)	≤ 0.25		≤ 0.25		0
*K. pneumoniae* Kpn20	32	R	8	R	4
*K. pneumoniae* 08Y70	32	R	2	S	16
*K. pneumoniae* 08Z37	16	R	4	I	4
*K. pneumoniae* 09A69	32	R	4	I	8
*K. pneumoniae* 09B51	8	R	1	S	8
*K. pneumoniae* 09B53	8	R	1	S	8
*K. pneumoniae* 09B61	32	R	4	I	8
*K. pneumoniae* 09B76	64	R	32	R	2
*K. pneumoniae* 09C12	64	R	16	R	4
*K. pneumoniae* 09C74	256	R	128	R	2
*K. pneumoniae* 09C77	32	R	4	I	8
*K. pneumoniae* 09D21	16	R	1	S	16
*K. pneumoniae* 10D60	16	R	2	S	8
*K. pneumoniae* 10F53	16	R	2	S	8
*K. pneumoniae* 10F74	32	R	8	R	4
*K. pneumoniae* 1‐57	16	R	2	S	8
*K. pneumoniae* 1‐60	16	R	4	I	4
*K. pneumoniae* 1‐61	128	R	128	R	0
*K. pneumoniae* 1‐70	32	R	4	I	8
*K. pneumoniae* 10I28	16	R	0.5	S	32
*E. coli*08Y79	8	R	2	S	4
*E. coli* 09B20	2	S	0.5	S	4
*E. coli* 09D25	2	S	≤ 0.25	S	≥ 8
*E. coli* 1‐37	1	S	≤ 0.25	S	≥ 4
*E. coli* 1‐47	16	R	4	I	4
*E. coli* 10F75	2	S	2	S	0
*E. coli* 2‐4	2	S	0.5	S	4

^a^EUCAST breakpoints 61: resistant MIC ≥ 8 mg·L^−1^; susceptible MIC ≤ 2 mg·L^−1^. (CLSI breakpoints are resistant MIC ≥ 4 mg·L^−1^; susceptible MIC ≤ 1 mg·L^−1^ 58).

### Crystal structure of VIM‐1:ML302F complex

**Figure 3:**
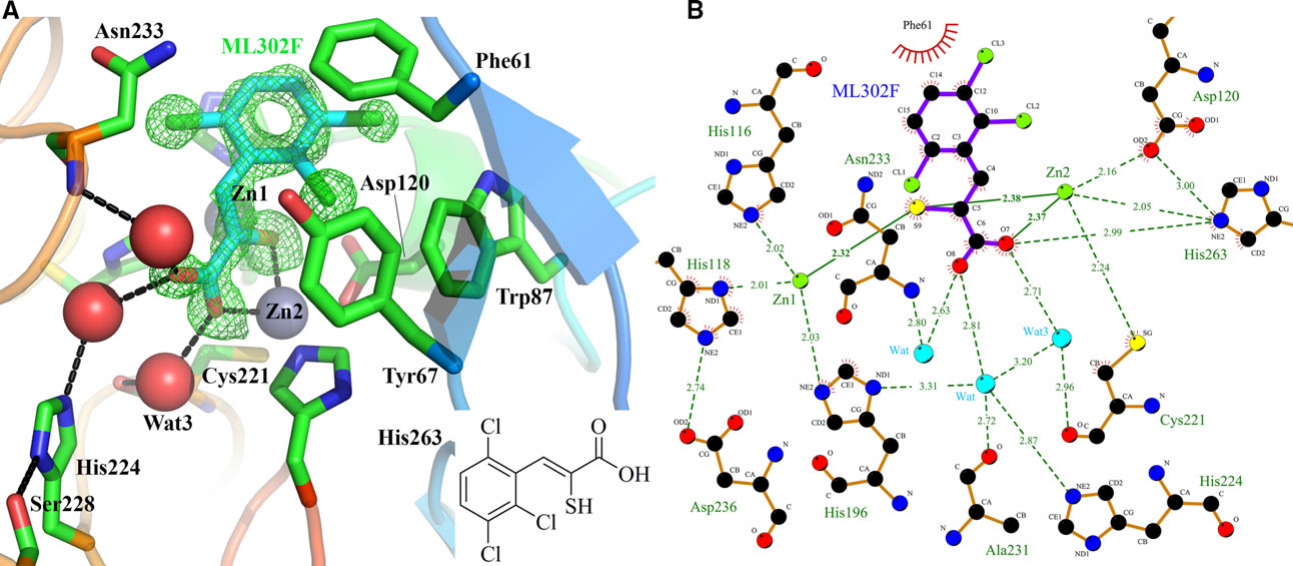
Interactions of thioenolate inhibitor ML302F with VIM‐1. (A) Structure of ML302F:VIM‐1 Complex. VIM‐1 main chain Cα atoms are colour ramped from blue (N terminus) to red (C terminus). Active site residues are rendered as sticks; zinc ions as grey spheres, water molecules as red spheres. Inhibitor carbon atoms are in cyan, side chain carbons in green. Other atom colours are as standard. Electron density shown is |*F*_o_| − |*F*_c_| Φ_calc_ contoured at 3 σ around inhibitor and calculated with the ligand omitted. Hydrogen bonding interactions are shown as dashed lines. Inset shows structure of ML302F. (B) Map of VIM‐1–ML302F interactions. Zinc ions are rendered in green; water molecules in cyan; other atom colours as standard. The figure was generated using ligplot 59.

We next sought to obtain structural information on the binding of ML302F to VIM‐1. An ML302F:VIM‐1 complex was obtained from a cocrystal, of the same symmetry as native VIM‐1, that yielded a diffraction dataset complete to 1.30 Å resolution. Two hundred and thirty‐four amino acids were built into this structure with four amino acids missing from the N terminus, and two from the C terminus. Five residues were built with alternative conformations. The Zn1 site was modelled with occupancy of 1.0, and the Zn2 site with occupancy 0.94. Initial electron density maps featured positive difference peaks in the active site that indicated the likely presence of bound ligand and into which ML302F could be readily fitted (Fig. 3A). ML302F was refined with occupancy 0.91 with a B‐factor (15.96 Å^2^) comparable to that of the protein (15.32 Å^2^).

Binding of ML302F (Fig. 3) manifests displacement of the di‐zinc ion bridging water molecule Wat1 by the inhibitor thiol, which intercalates between the two zinc ions (distances 2.32 and 2.38 Å from Zn1 and Zn2 respectively). The inhibitor carboxylate displaces Wat2 to make an electrostatic interaction with Zn2 (distance 2.37 Å), with the consequence that Zn2 is fivefold coordinated in a trigonal bipyramidal geometry. Water‐mediated interactions reminiscent of those observed in carbapenem complexes (see below) connect one oxygen atom of the inhibitor C1 carboxylate to the Ala231 carbonyl and His224 Nδ1, and, via an additional water molecule, to the Asn233 backbone amide. An additional water molecule, Wat3, connects the Zn2‐bound oxygen to the Cys221 carbonyl. The trichlorine substituted phenyl ring of ML302F makes a π‐stacking interaction with Phe61 at the base of loop L3, with one of the halogen atoms making a chlorine‐π interaction with Trp87.

### Interactions of VIM‐1 with hydrolysed meropenem

Although recent progress has been made in structural characterization of the interactions of B1 MBLs with hydrolysed β‐lactam substrates 33, 34, 35, 36, to date there is no reported information on how VIM enzymes bind their β‐lactam substrates. Given the clinical importance of the VIM enzymes, and their lack of Lys224, a residue likely crucial in the interaction of most other B1 MBLs with the C2/C3 carboxylate group of β‐lactams, information regarding the mode of β‐lactam binding to VIM‐1 is important both to understanding the mechanism of β‐lactam hydrolysis and design of inhibitors. Hence, to investigate the interactions of VIM‐1 with β‐lactams, we soaked native VIM‐1 crystals with meropenem with the aim of obtaining structural information on enzyme‐bound species.

**Figure 4:**
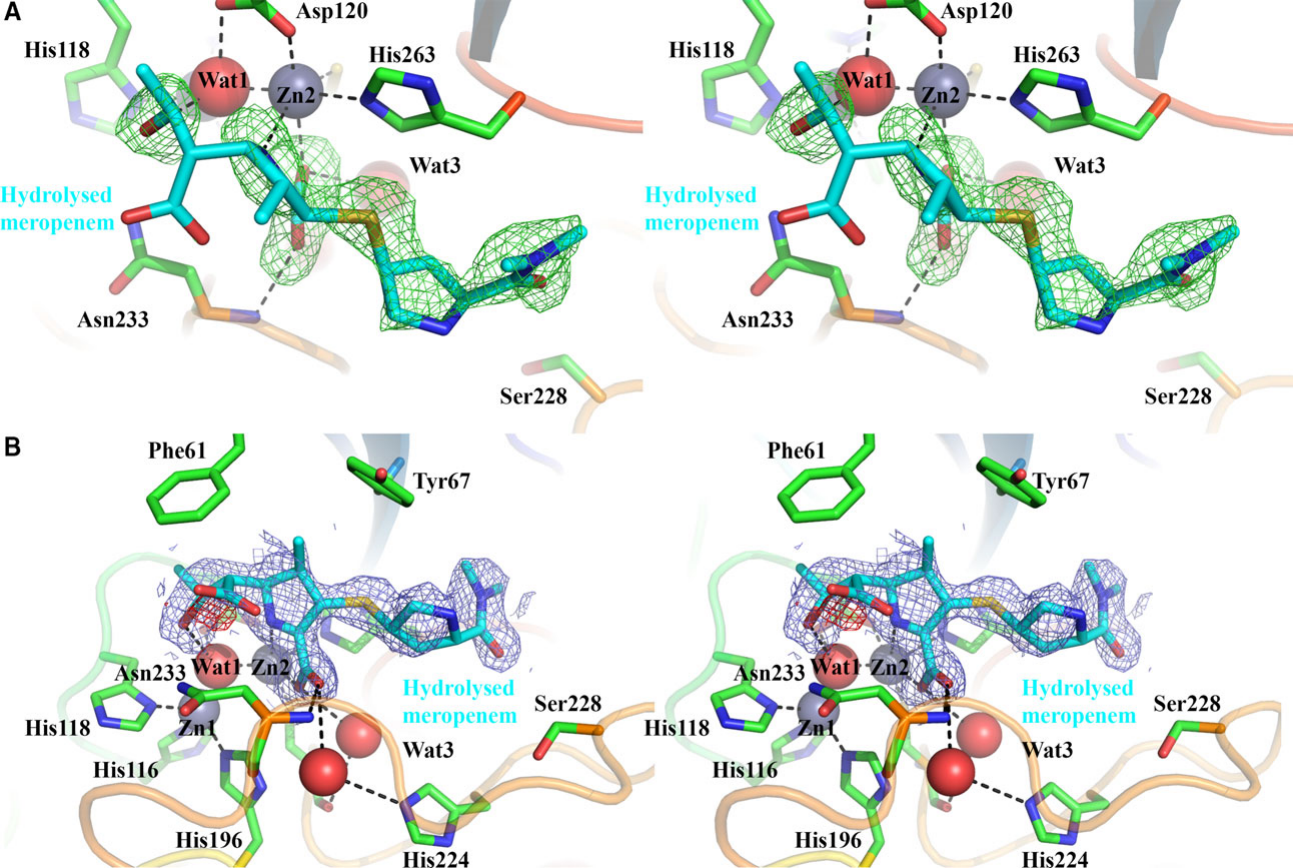
Electron density maps for hydrolysed meropenem bound to VIM‐1. (A) Stereoview of |*F*_o_| − |*F*_c_| Φ_calc_ electron density (green; calculated with ligand omitted) contoured at 2.5 σ around hydrolysed meropenem (cyan). Note the position of the S atom out of the plane of the pyrroline ring. (B) Stereoview of 2|*F*_o_| − |*F*_c_| Φ_calc_ electron density (blue) and |*F*_o_| − |*F*_c_| Φ_calc_ electron density (negative peak red; both calculated with ligand included) contoured at 1.0 and 3.0 σ respectively, around hydrolysed meropenem (cyan). Note the presence of only very limited negative density close to the C7 carboxylate. Active site residues are rendered as sticks; zinc ions as grey spheres, water molecules as red spheres. Other atom colours are as standard.

Inspection of *F*_o_ − *F*_c_ difference density maps calculated from a diffraction data set collected from a native VIM‐1 crystal after overnight exposure to meropenem powder revealed positive density peaks into which hydrolysed meropenem could be refined (Fig. 4A). This yielded a structure for a complex to a resolution of 2.20 Å. The structure contains 232 amino acids with five residues missing from the N terminus and three from the C terminus. Four residues were built with alternative side chain conformations. Zinc ions were refined in the Zn1 (occupancy 1.0) and Zn2 (occupancy 0.87) sites, with a third zinc ion (occupancy 0.55) involved in crystal contacts at the interface of VIM‐1 molecules in two adjacent asymmetric units. Notably, Zn2 presented a higher than average B‐factor (33.67 Å^2^ compared to 13.18 Å^2^ for Zn1).

**Figure 5:**
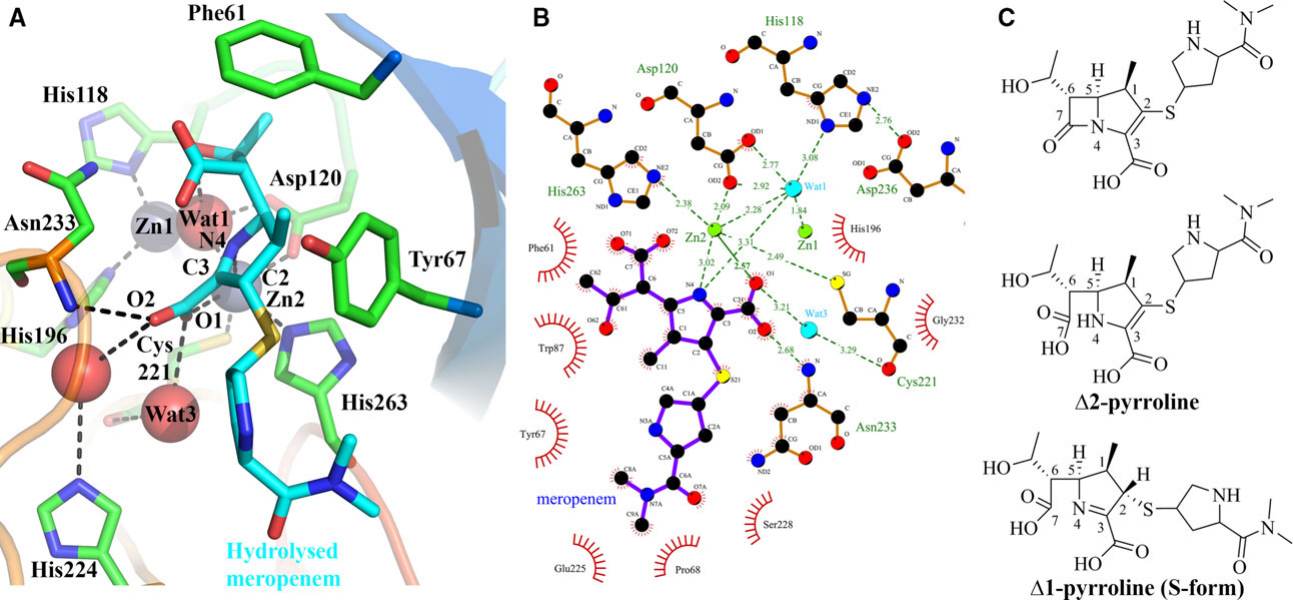
Interactions of hydrolysed meropenem with VIM‐1. (A) Interactions made by hydrolysed meropenem with VIM‐1 active site. Hydrogen bonds are shown as dashed lines. (B) Map of VIM‐1 interactions with hydrolysed meropenem. Zinc ions are rendered in green; water molecules in cyan; other atom colours as standard. (C) Structure of intact and hydrolysed meropenem in Δ^2^ (B) and Δ^1^ (C) pyrroline forms.

Compared to the VIM‐1:ML302F complex, binding of hydrolysed meropenem to VIM‐2 is less well defined – in addition to the lower resolution, elevated B‐factors were observed for the hydrolysed meropenem ligand (overall B‐factor of 39.13 Å^2^ compared to 16.99 Å^2^ for the protein main chain). Nevertheless, refining the ligand at full occupancy yielded a real‐space correlation coefficient of 0.85 and real‐space R‐value of 0.25; parameters that indicate acceptable agreement of observed and calculated electron densities for bound ligand 37. This is confirmed by visual inspection; omit (|*F*_o_| − |*F*_c_| Φ_calc_; Fig. 4A) electron density maps define the positions of key elements of hydrolysed meropenem – the dihydropyrrole ring and associated N4 nitrogen and C3 carboxylate groups, and the S atom and pyrrolidine ring of the C2 substituent. Appropriate ligand placement is also evident based on inspection of 2|*F*_o_| − |*F*_c_| Φ_calc_ and associated difference (|*F*_o_| − |*F*_c_| Φ_calc_) maps (Fig. 4B), with the latter providing no strong negative peaks indicating substantial errors in ligand placement. However, omit electron density is weaker for the methyl group attached to C1, the carbapenem C6 hydroxyethyl group and, in particular for the C7 carboxylate that is formed on hydrolysis of the β‐lactam ring. This was positioned after trial refinements in several alternative orientations, including in the zinc‐bridging position, with the final structure representing the mode of binding that minimised overly close contacts with Zn ions and coordinating residues, such as Asp120, and steric clashes of the C6 hydroxyethyl with the adjacent side chains of Phe61 and Trp87. Hence, as in the uncomplexed VIM‐1 structure, Wat1 is closer to Zn1 (1.84 Å) than Zn2 (2.28 Å, Fig. 5A,B), and the presence of hydrolysed meropenem has little effect on the Zn–Zn separation distance (3.52 Å compared to 3.62 Å in the uncomplexed structure).

Carbapenems contain a 4 : 5 fused β‐lactam ring system with a double bond between C2 and C3 in the five‐membered pyrroline ring. This results in two possible tautomeric forms (Δ^1^ and Δ^2^ pyrroline) for the product resulting from hydrolysis of the β‐lactam ring (Fig. 5C 10, 38). In intact meropenem the C2/C3 carbon atoms are sp2 hybridised, due to the double bond between them, and are coplanar with the C2‐linked S atom. This configuration can be retained after β‐lactam hydrolysis. However, in ring‐opened carbapenems tautomerization of the enamine double bond can occur, giving rise to the Δ^1^ (imine) form with a double bond between C3 and N4. Our experimental data, at 2.2 Å resolution, cannot permit unambiguous assignment of the tautomeric form present. However, experimental difference electron density maps (Fig. 4A) indicate the S atom as lying out of the plane of the pyrroline ring, suggesting that the meropenem C2 is sp3, rather than sp2, hybridized. In consequence, we have refined the ligand in the Δ^1^ (imine), rather than the Δ^2^, tautomer, and in the (S) (β), rather than (R) (α) stereochemistry (Figs 4 and 5).

These limitations notwithstanding, the comparatively strong electron density observed for elements of the ligand interacting with the MBL metal centre (N4 nitrogen and C3 carboxylate groups) defines key interactions of VIM‐1 with hydrolysed meropenem (Fig. 5A,B) that involve the Zn2 metal ion. Zn2 displays octahedral co‐ordination, contacting O1 of the substrate C3 carboxylate (in the Wat2 position of the native active site, 2.57 Å) and N4 of the hydrolysed β‐lactam‐derived amine. However, the Zn2–N4 interaction is apparently weak (3.02 Å) compared to the equivalent contacts in other complexes of MBLs with hydrolysed β‐lactams (possibly reflecting more facile release of hydrolysis product by VIM‐1). The C3 carboxylate is also positioned to make additional interactions with the enzyme, both directly (via its O2 atom and the backbone amide of Asn233; 2.68 Å) and indirectly (between O2 and the imidazole side chain of His224 via an additional water, and between O1 and the main chain carbonyl of Cys221 via a water molecule in the Wat3 position). Wat3 thus plays similar roles in carboxylate binding in both the meropenem and ML302F complex structures.

## Discussion

Clinically relevant MBLs are largely contained within the B1 subclass. The VIM enzymes are among the most important and are noted for their wide bacterial species and geographical distribution and diversity (60 variants at the time of writing). Variation in VIM enzymes is notable as it occurs at two positions, 224 and 228, that are proposed to interact with β‐lactam substrates in other MBLs 23, raising the possibility that different variants bind ligands (substrates or inhibitors) in different ways. Different variants feature Tyr, His or Leu at position 224 and Arg, Ser or Leu at 228. Thus, while VIM‐2 Arg228 is proposed to contact the substrate carboxylate as a functional equivalent of Lys224 in other B1 MBLs 24, 39, other VIM variants can be expected to interact with this key β‐lactam functional group in different ways.

**Figure 6:**
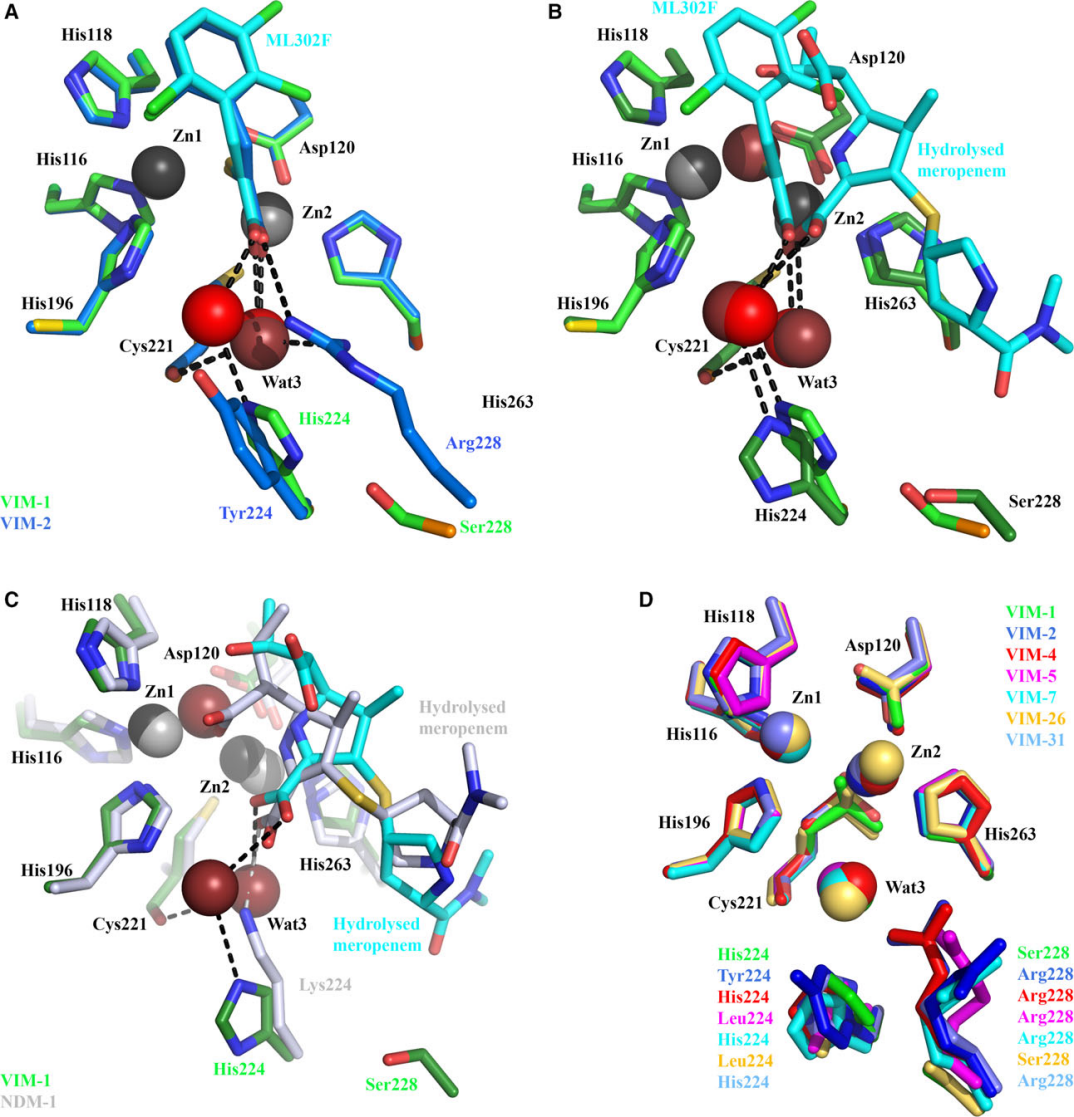
Importance of Cys221‐bound water to ligand binding by VIM‐1. (A) Mode of ML302F binding to VIM‐1 (green) and VIM‐2 (pdb 4PVO 30; blue). Wat3 in VIM‐2 structure is in dark red. (B) Overlay of ML302F and hydrolysed meropenem complexes with VIM‐1. The active site view of the ML302F complex is shown in lighter, and meropenem in darker, shades. (C) Overlay of hydrolysed meropenem binding to VIM‐1 (darker colours) and NDM‐1 (pdb 5N0H 35, 60; lighter colours). (D) Overlay of available VIM crystal structures (details as Fig. 1) showing conserved location of Wat3. Colours are as specified. Note how Wat3 mediates interactions of both substrate and inhibitor carboxylate groups with the Cys221 backbone carbonyl, and substitutes for NDM‐1 Lys224 in binding the carboxylate of hydrolysed meropenem (Panel C).

Binding of the thioenolate inhibitor ML302F to VIM‐1 gives insight into how different VIM variants might interact with both inhibitors and substrates. Previous studies 30, 40 identified ML302F as a low micromolar inhibitor of both VIM‐1 and VIM‐2 that potentiates meropenem activity against VIM‐expressing laboratory and clinical Enterobacteriaceae in susceptibility testing and promotes survival of infected insect (*Galleria*) larvae 40. The current work extends these observations by demonstrating activity of the ML302F–meropenem combination against a wider panel of VIM‐1 producing clinical strains. Moreover, when considered with the existing VIM‐2:ML302F structure 30, our structure of the VIM‐1:ML302F complex enables, for the first time, direct comparison of how the same ligand is bound by two different VIM variants (Fig. 6A).

ML302F is almost identically positioned in the two structures. Similar to VIM‐1 binding (above), the ML302F thiol intercalates between the two zinc ions and the carboxylate interacts with the VIM‐2 Zn2 ion. In addition, the ML302F carboxylate makes direct (hydrogen bond) contact with the guanidinium side chain of VIM‐2 Arg228. In VIM‐1, the equivalent oxygen of the ML302F carboxylate makes a water‐mediated interaction with His224. However, in both complexes the ML302F Zn2‐bound oxygen atom also interacts with the backbone carbonyl of the Zn2 ligand Cys221 via a water molecule, Wat3. Wat3 adopts near‐identical positions in the VIM‐1 and VIM‐2 complexes, implying a conserved role in substrate/inhibitor carboxylate binding. This conclusion is further supported by near‐identical interactions in complexes of VIM‐2 with cyclic boronate 41 and triazolylthioacetamide 42 inhibitors, that similarly involve Wat3 in binding carboxylate.

Involvement of Wat3 in ML302F binding by VIM‐1 and VIM‐2, along with the similarity between ML302F binding to VIM‐2 and interactions of hydrolysed β‐lactams with other B1 MBLs 30, suggests that VIM enzymes might make equivalent contacts with β‐lactams. Indeed, comparison of our ML302F and meropenem VIM‐1 complexes indicates that the oxygen atoms of the ML302F C1 and meropenem C3 carboxylates adopt near‐identical positions and, importantly, make equivalent interactions with the VIM‐1 active site (Fig. 6B). In both cases the Zn2‐bound oxygen contacts the Cys221 backbone carbonyl via Wat3, supporting involvement of Wat3 in β‐lactam, as well as inhibitor, binding by VIM‐1 and, by implication, other VIM enzymes. The other carboxylate oxygen hydrogen bonds, via a water molecule, to His224, thus providing a mechanism for involvement of this residue in substrate binding, although it is too distant (> 5.7 Å) from the meropenem carboxylate to make a direct interaction. Increases in *K*_M_ values (i.e. reduced affinity) for carbapenem and some cephalosporin substrates for VIM‐26 (VIM‐1 His224Leu) compared to VIM‐1 25 also support the involvement of VIM‐1 His224 in β‐lactam binding. However, the fact that VIM‐26 retains catalytic activity shows that a residue at position 224 able to make electrostatic interactions with substrate is not essential to activity of VIM enzymes.

Until now, structures of complexes of VIM MBLs with hydrolysed β‐lactams have been elusive. However, these are available for the B1 MBL NDM‐1, permitting comparison of meropenem binding to VIM‐1 with β‐lactam binding to NDM‐1. In such structures, the C2/C3 carboxylates of hydrolysed penicillins 34, 35, 36, cephalosporins 33 and carbapenems 35, 43 contact the NDM‐1 Zn2 metal ion, the backbone amide of Asn233 and the terminal amino group of Lys224. Comparing hydrolysed meropenem binding to VIM‐1 and NDM‐1 (Fig. 6C), in each case the C2 carboxylate adopts similar orientations and makes equivalent interactions with Zn2 and the Asn233 backbone amide; but the two complexes differ in the interactions made by the Zn2‐bound carboxylate oxygen, which in NDM‐1 hydrogen bonds to Lys224 and in VIM‐1 to the Cys221 backbone carbonyl via Wat3. Wat3 thus enables VIM‐1, and by implication other VIM enzymes, to replicate interactions with substrates made by Lys224 in other B1 MBLs. The importance of these to β‐lactam binding and hydrolysis is evidenced by the profound loss of activity observed in Lys224 mutants of other B1 enzymes 44, 45, 46. Thus, in both of the structures presented here, as well as the ML302F:VIM‐2 complex, the water molecule Wat3 connects the carboxylate of hydrolysed β‐lactam/inhibitor to the Cys221 backbone carbonyl. These observations indicate that Wat3 enables VIM enzymes to replicate the role of Lys224 in ligand binding by other subclass B1 MBLs, while simultaneously accommodating sequence variations at positions 224 and 228. To investigate this hypothesis, we inspected crystal structures of VIM MBLs (Fig. 6D). This analysis identified water molecules in the Wat3 position in all available VIM variant structures and their complexes in the active (binuclear) form. We thus propose that this active site water molecule, positioned by the backbone of the invariant Zn2 ligand Cys221, enables different VIM variants to make productive interactions with substrate even where, as in VIM‐1, direct contact between the C2/C3 carboxylate and protein is impossible.

Mechanistic interpretation of the complex with bound meropenem is limited by both the resolution of our data, and the weak electron density for the C6 hydroxyethyl and C7 carboxylate groups. The situation is further complicated by the likelihood that several species are likely to be present, given that carbapenem breakdown in solution can yield a mixture of hydrolysis products, that is, both the Δ^1^ (R and S stereoisomers) and Δ^2^ tautomers 47 and that trapped complexes may thus represent either species present on the late stages of the reaction pathway or rebinding of highest affinity products present in solution. However, we note that in the present structure the well‐defined meropenem N4 atom lies more distant (3.02 Å) from Zn2 than in other such complexes with B1 MBLs, where this is typically a tight interaction (≤ 2.40 Å). When considered together with our inclusion of a zinc‐bridging water molecule in our final model, (which is consistent with available structures for NDM‐1–penicillin and cephalosporin‐derived complexes 33, 34, 35, 36), one possible interpretation is that our complex could represent a stage in product release where water has displaced the C7 carboxylate from the Zn‐bridging position. This would indicate that release of product from the MBL active site is not a single step process, and that interactions of β‐lactams around the Zn2 site persist longer than those involving Zn1.

Overall, our work provides the first structural description of VIM‐1, an MBL of particular relevance to the growing problem of carbapenem‐resistant Enterobacteriaceae. Our structures of VIM‐1–meropenem and inhibitor complexes establish how VIM MBLs accommodate variation at residues 224 and 228, without loss of activity, and provide new information on carbapenem hydrolysis, specifically related to product release. Identification of interactions, involving carboxylate groups of small molecule ligands and the invariant water molecule Wat3, that are common to VIM‐1, VIM‐2 and, we infer, other VIM variants, will aid in structure‐based rational development of effective inhibitors for the full range of these heterogeneous and clinically important enzymes.

## Experimental procedures

### Materials

General laboratory reagents were purchased from Sigma (Poole, UK) or VWR (Lutterworth, UK) and were of analytical grade. Inhibitor ML302F was synthesized as previously described 30. Meropenem was a gift from AstraZeneca (Macclesfield, UK).

### Cloning, expression and purification

Full‐length VIM‐1 with a C‐terminal 6His tag was expressed and purified largely according to previously published procedures 48 using the pOPINE T7 plasmid 49 containing a synthetic codon optimized gene. Modifications are detailed below. Protein was expressed in *E. coli* Rosetta 2 (DE3) (Merck, Watford, UK) with cells (500 mL in 2 L conical flasks) grown in Terrific Broth autoinduction media (Formedium, Hunstanton, Norfolk, UK) supplemented with 100 μg·mL^−1^ ampicillin. Cultures were grown at 37 °C shaking at 160 r.p.m. for 8 h; the temperature was subsequently lowered to 25 °C for overnight growth. Cultures were harvested by centrifugation (7 200 ***g***, 30 min, 4 °C) and pellets from 3 L cells resuspended in 200 mL of buffer A (50 mm Tris pH 7.5, 500 mm NaCl, 30 mm imidazole) supplemented with 20 μL of 5 kU benzonase (Sigma Aldrich, Poole, UK), 0.02% Tween 20 (Sigma) and two EDTA‐free protease inhibitor cocktail tablets (Roche, Burgess Hill, UK). A Constant Cell Disruption System (Constant Systems, Daventry, UK) was used to lyse homogenized cells at 25 kpsi, the lysate was cleared by centrifugation (1 h, 38 500 ***g***, 4 °C). 6His‐tagged VIM‐1 was purified on a 5‐mL HisTrap HP column (GE Life Sciences, Little Chalfont, UK) using a 0–500 mm imidazole gradient, as described 48. Protein obtained from this single‐step purification was estimated as being over 98% pure as adjudged by SDS/PAGE. Fractions containing VIM‐1 were pooled and concentrated by centrifugation (10 kDa molecular weight cut off Amicon Ultra‐15 (Merck). Protein used to obtain the di‐zinc VIM‐1 structure was further purified by size exclusion (Superdex‐200) as described 48; samples used to obtain other structures were used without further purification.

### Crystallization, data collection and structure solution

Crystallization screening used commercial sparse matrix screens (Molecular Dimensions, Newmarket, UK; Qiagen, Manchester, UK) dispensed via a Hydra 96 microdispenser (Robbins Scientific, Sunnyvale, CA, USA). A Honeybee X8 (Digilab, Hopkinton, MA, USA) robot was used to set up crystallization trials in 96‐well sitting drop plates (CrystalQuick, Greiner, Stonehouse, UK) with drops containing 0.1 μL of protein and 0.1 μL of reservoir solution and a total reservoir volume of 95 μL. Crystals were stored and monitored at 21°C using a Rock Imager 1000 system (Formulatrix, Bedford, MA, USA). The di‐zinc VIM‐1 structure was solved using a crystal obtained from protein concentrated to 15 mg·mL^−1^ (containing 7% glycerol) and crystallized under conditions (0.05 m MgCl_2_, 0.03 m CaCl_2_, 0.1 m Morpheus buffer 3 pH 8.5, 13.5% w/v poly(ethylene glycol) 3350, 9.5% w/v poly(ethylene glycol) 1000 and 12.5% w/v MPD) obtained by optimization of an initial hit from the Morpheus screen 50. This crystal was obtained from a drop set up by hand in 24‐well hanging drop format using 1 μL protein and 1 μL reservoir condition and a total reservoir volume of 500 μL.

Other VIM‐1 structures were obtained from protein concentrated to ~ 23 mg·mL^−1^ containing 10% glycerol and supplemented with 100 μm ZnCl2, 2 mm tris(2‐carboxyethyl)phosphine (Thermo Scientific, Loughborough, UK). Crystals of the VIM‐1:ML302F complex were obtained from 0.02 m Na‐formate, 0.02 m NH_4_‐acetate, 0.02 m Na_3_‐citrate, 0.02 m NaK‐tartrate, 0.02 m Na‐oxamate, 0.1 m Morpheus Buffer 1 pH 6.5, 12.5% w/v poly(ethylene glycol) 3350, 12.5% w/v poly(ethylene glycol) 1000 and 12.5% w/v MPD. Crystallization drops were set up by hand in 96‐well MRC sitting drop plates (Molecular Dimensions) using commercial screens with 0.5 μL of protein and 0.5 μL reservoir solution and a total reservoir volume of 50 μL. Experiments were incubated at 20 °C. Excess inhibitor was added to the drop in the form of powder. The structure of the VIM‐1–meropenem complex was obtained from an overnight soak of crystals obtained from a different Morpheus condition (0.1 m MOPS/HEPES‐Na pH 7.5, 12.5% w/v poly(ethylene glycol) 1000, 12.5% w/v poly(ethylene glycol) 3350, 0.03 m CaCl_2_, 0.03 m MgCl_2_ and 12.5% v/v MPD) with excess substrate powder. Crystals were snap‐frozen in liquid nitrogen prior to transportation for diffraction data collection.

Crystallographic data were collected at 100 K on beamlines of Diamond Light Source (DLS; Didcot, UK) using Pilatus 6M‐F detectors. Diffraction data were integrated with xds and scaled using aimless as part of the ccp4 software suite 51, 52, 53. Phases were solved by molecular replacement in phaser 54 using either VIM‐4 (PDB 2WGH 29) or previously determined VIM‐1 structures as search models. Rounds of refinement were carried out using phenix 55 with manual rebuilding in coot 56. molprobity 57 was used for structure validation as part of the phenix suite. pymol (www.pymol.org) was used to generate figures. Data collection and refinement statistics, together with Protein Data Bank (pdb) accession codes, are presented in Table 1.

### Determination of minimal inhibitory concentrations

Twenty clinical strains of *K. pneumoniae* and seven clinical strains of *E. coli*, originating from clinical specimens from Spain, were confirmed for the presence of VIM‐1 by PCR assay using the primers VIM‐F (5′‐CCG ACA GTC ARC GAA ATT CCG‐3′) VIM‐R (5′‐CTA CTC RRC GAC TGA GCG ATT‐3′). MIC values were determined by broth microdilution, in triplicate, in cation‐adjusted Mueller–Hinton broth (Sigma) according to the Clinical Laboratory Standards Institute (CLSI) guidelines 58. Experiments were carried out in microtitre plates (Corning) containing the medium plus meropenem and inhibitor ML302F [dissolved in DMSO and added to the wells with a final concentration of 10 μg·mL^−1^ (0.1% DMSO)]. Plates were incubated overnight at 37 °C for 18–24 h and absorbance at 600 nm read using a Polarstar Omega (BMG LabTech, Aylesbury, UK) plate reader.

## Conflicts of interest

The authors declare no conflicts of interest.

## Author contributions

JS, RJO, CJS and TRW designed the study. RS and AV overexpressed, purified and crystallized VIM‐1; RS, MK, PH and JS collected X‐ray data and solved and refined structures. SSB synthesized compound ML302F. JMT identified and characterized clinical Enterobacteriaceae. RS conducted MIC experiments. RS, PH, JS, JB and MAM analysed and compared structures. JS wrote the paper with contributions and approvals from all authors.
